# Righting response and electrocardiographic evaluation of juvenile tambaqui (*Colossoma macropomum*) (Cuvier, 1818) exposed to immersion baths with different concentrations of bushy lippia essential oil (*Lippia alba*)

**DOI:** 10.1371/journal.pone.0314458

**Published:** 2025-02-03

**Authors:** Thaysa de Sousa Reis, Clarissa Araújo da Paz, Daniella Bastos de Araújo, Luciana Eiró Quirino, Yris da Silva Deiga, Tays Mata Câmara, Moisés Hamoy

**Affiliations:** Laboratory of Pharmacology and Toxicology of Natural Products, Biological Science Institute, Federal University of Pará, Belém, Pará, Brazil; Kerman University of Medical Sciences, ISLAMIC REPUBLIC OF IRAN

## Abstract

In recent years, the use of natural products as alternatives to synthetic anesthetic agents has gained notoriety in aquaculture. Among the essential oils studied, *Lippia alba* has attracted attention due to its diverse pharmacological properties, including sedative and anesthetic effects. This study aims to evaluate the anesthetic activity of *Lippia alba* essential oil (LAEO) to propose a therapeutic window for its use in tambaqui (*C*. *macropomum*). In this research, juvenile tambaqui (10.9 ± 5.9g, n = 90) were submitted to anesthetic baths with LAEO at concentrations of 80–140 μL.L^-1^ for behavioral assessment and electrophysiological recordings. The findings showed that all concentrations were effective in inducing the loss of posture reflex and its subsequent reversible return. However, electrocardiographic recordings at concentrations of 120 and 140 μL.L^-1^ revealed alterations in the graph elements, indicating more intense bradycardia and atrioventricular block during anesthetic induction. Nevertheless, during the anesthetic recovery period, cardiac normality was restored at all tested concentrations, although more slowly at the highest concentration (140 μL.L^-1^). Thus, we conclude that this oil is safe for providing short-term anesthesia at concentrations between 80 and 100 μL.L^-1^ in *C*. *macropomum* specimens.

## Introduction

There is a growing number of studies investigating the application of natural products such as essential oils and their active substances as potential replacements for synthetic anesthetics in aquaculture [1,2]. *Lippia alba*, an aromatic plant from the Verbenaceae family widely cultivated in Brazil and commonly known as “lemon balm” has gained attention due to its diverse pharmacological properties [3,4]. The anesthetic, antifungal, and antimicrobial activities of *L*. *alba* essential oil are well-documented by various authors [5–7]. The sedative and anesthetic actions of *L*. *alba* essential oil are directly related to the compounds citral (geranial and neral) and linalool, possibly through the modulation of the GABAergic system [8–10].

In light of this, the applicability of this essential oil as an anesthetic agent has been investigated in various fish species. A previous study reinforces the efficacy of this oil, noting that in addition to its anesthetic activity, it also reduces stress in tambacu (*Piaractus mesopotamicus* x *Colossoma macropomum*) during handling and transportation [11]. Furthermore, this essential oil has been associated with antioxidant effects that are crucial during periods of hyperoxia and hypoxia occurring during fish transport [12]. However, high doses of *L*. *alba* can lead to toxic effects, such as osmoregulatory changes and alterations in cortisol and glucose levels [13,14]. Therefore, it is evident that investigating different concentrations of this essential oil and its secondary effects on the animal is of significant importance.

There are numerous studies in the literature employing *L*. *alba* essential oil as an anesthetic agent in *C*. *macropomum*. In the study conducted by Souza et al. (2018) [15], the anesthetic efficacy of *L*. *alba* essential oil, chemotype linalool, was particularly evident, demonstrating effective sedation and anesthesia without compromising the recovery period. Additionally, a prior study establishes an anesthetic protocol for tambaqui using *L*. *alba* essential oil [5]. Although the anesthetic efficacy of *L*. *alba* is well-known, its systemic effects on *C*. *macropomum*, particularly its association with cardiac activity to delineate a safety margin for the use of this anesthetic, are not well understood.

Therefore, considering the potential properties of this essential oil as an anesthetic for fish and the lack of detailed information on its effects on the cardiac function of these animals, this study aims to provide an evaluation of the electrocardiogram of juvenile tambaqui subjected to anesthetic baths containing *L*. *alba* essential oil, in order to examine the risks and establish an appropriate therapeutic window for its use as an anesthetic.

## Materials and methods

### Experimental animals

The fish used for this research were 90 juveniles tambaquis, *Colossoma macropomum*, which were kept in aquariums (500 L) at the Experimental Animal Facility of the Laboratory of Pharmacology and Toxicology of Natural Products at the Federal University of Pará (LFTPN-ICB-UFPA). The room had a controlled temperature (25 to 28°C) and a 12-hour light: 12-hour dark photoperiod, and the fish were fed commercial feed (32% protein) twice daily until satiation. Simultaneously, daily tank cleaning was performed through siphoning, and approximately 20% of the tank water was replaced with water from the same source. During the 15-day acclimation period, water quality variables such as temperature (°C), hydrogen potential (pH), dissolved oxygen (DO), ammonia (NH3+), and total hardness were monitored and maintained within the following values: Temp 26.8°C; pH 7.5; DO > 5.0 mg.L^-1^; NH3+ 0.01 mg.L^-1^; total hardness 70 NTU. This project was approved by the Ethics Committee on Animal Use of the Federal University of Pará (CEUA/UFPA) under protocol number 1024010224.

### Acquisition, preparation, and preservation of the drug

Essential oil of *Lippia alba*, produced by the company Legeé óleos essenciais (Brasial–SP), with chromatographic data performed at the Laboratory of the University of Santa Cruz do Sul using a Gas Chromatograph with Mass Detector—Agilent Model MSD 5977B, showed the components described in Table 1. The essential oil of *Lippia alba* was diluted to 10% in 70% alcohol for use during the experiment.

**Table 1:** Chromatographic profile of *Lippia alba* essential oil, used in this study.

Identification	Relative area percentage (%)	Similarity (%)	Retention time (%)
**Linalool**	66,16	98,39	21,18
**Eucalyptol**	8,44	98,74	16,05
**Caryophyllene**	6,08	98,71	52,73
**β-Copaene**	4,14	98,85	56,06
**β-Elemene**	2,63	96,71	50,78
**γ-Elemene**	2,60	96,56	53,42
**Sabinene (β-Thujene)**	1,54	97,80	12,50
**α-Pinene**	1,38	98,44	10,67
**Humuleno**	1,01	97,16	54,78
**Caryophyllene oxide**	0,81	97,95	60,09
**Germacrene B**	0,62	97,60	59,24
**Copaene**	0,59	96,44	49,58
**δ-Cadinene**	0,52	95,46	57,70
**Valence**	0,36	93,09	55,00
**β-Bourbonene**	0,35	89,27	50,22
**β-Myrcene**	0,34	89,95	13,34
**D-Limonene**	0,29	91,50	15,84
**(+)-epi-Bicyclosesquiphellandrene**	0,27	88,34	50,64
**Bicyclosesquiphellandrene**	0,27	87,85	50,64
**β-Copaen-4α-ol**	0,26	89,86	65,01
**Linalool oxide**	0,25	91,67	18,83
**α-Muurolene**	0,24	95,81	56,89
**γ-Cadinene**	0,23	95,55	55,80
**Ylangenal**	0,22	93,04	62,60
**Elemene isômero**	0,21	93,64	56,72
**trans-Linalool oxide (furanoid)**	0,19	88,87	20,12

Indication of relative percentage (%), similarity (%), retention time (%), and registration in the Chemical Abstracts Service (CAS).

### Experiment 1—Description of the behavioral experiment

For the behavioral evaluation, the animals (10.9 ± 5.9g) were subjected to immersion baths with *Lippia alba* essential oil (LAEO). The groups were divided according to the following concentrations: a) 80 μL.L^-1^ group; b) 100 μL.L^-1^ group; c) 120 μL.L^-1^ group; d) 140 μL.L^-1^ group. Specific concentrations of this essential oil were tested by Silva et al. (2019). In contrast, the present study examines a broader range of concentrations to explore a more continuous gradient of effects.

For each treatment, n = 9 animals were used, totaling 36 animals. The behavioral assessment was conducted over 5 minutes for induction and recovery of anesthesia, evaluating the time to loss of posture reflex and recovery of posture reflex, respectively.

### Experiment 2—Description of the electrophysiological experiment (electrocardiogram ECG)

The *C*. *macropomum* were randomly distributed into the following treatments: a) control group (baseline activity recording); b) vehicle group (only the vehicle was used to assess interference in the experiment at 1,400 μL.L^-1^) c) 80 LAEO group; d) 100 μL.L^-1^ LAEO group; e) 120 μL.L^-1^ LAEO group; f) 140 μL.L^-1^ LAEO group. All cardiac activity recordings lasted for 5 minutes. For each recording, n = 9/treatment were used, totaling 54 animals. The analysis of electrocardiographic recordings was performed during induction and during anesthetic recovery.

### Acquisition of the electrocardiogram (ECG)

For the analysis and monitoring of cardiac function, electrodes were made of 925 silver with a diameter and length of 0.3 mm and 10 mm, respectively, in a non-conjugated manner. The position used for fixing the reference electrode followed the indication of the cardiac vector and was fixed ventrally 2 mm before the end of the left opercular cavity (reference electrode). The recording electrode was inserted 2 mm before the end of the right opercular cavity (ventral). After the insertion of the electrodes, they were connected to a high-impedance amplifier (Grass Technologies, Model P511) for the acquisition of electrocardiographic records. With these, the following parameters were analyzed: heart rate (bpm), amplitude (mV), QRS complex duration (ms), and R-R (ms), P-Q (ms), and Q-T (ms) intervals.

### Recording and analysis of records

The electrodes were connected to a digital data acquisition system through a high-impedance differential input amplifier (Grass Technologies, Model P511), configured for filtering from 0.3 to 300 Hz and amplification of 2000X, monitored by an oscilloscope (ProteK, Model 6510). The records were continuously recorded and digitized at a rate of 1 KHz on a computer with a data acquisition board (National Instruments, Austin, TX), and stored on a hard drive for further processing using specialized software (LabVIEW express). Record analysis was performed using Python version 2.7. For mathematical processing, the Numpy and Scipy libraries were used, and for graph creation, the Matplotlib library. The graphical interface was developed using the PyQt4 library (Souza-Monteiro et al., 2015). The amplitude graphs, in turn, highlighted the potential differences between the reference and recording electrodes. The signals from the records were obtained at 1000 samples per second.

### Statistical analysis

After verifying the assumptions of normality and homogeneity of variances using the Kolmogorov-Smirnov and Levene tests, respectively, comparisons of mean power values were conducted using one-way ANOVA, followed by Tukey’s test. GraphPad Prism^®^ 8 software was used for the analyses, and a significance level of *p < 0.05, **p < 0.01, and ***p < 0.001 was considered statistically significant in all cases.

## Results

**Fig 1:**
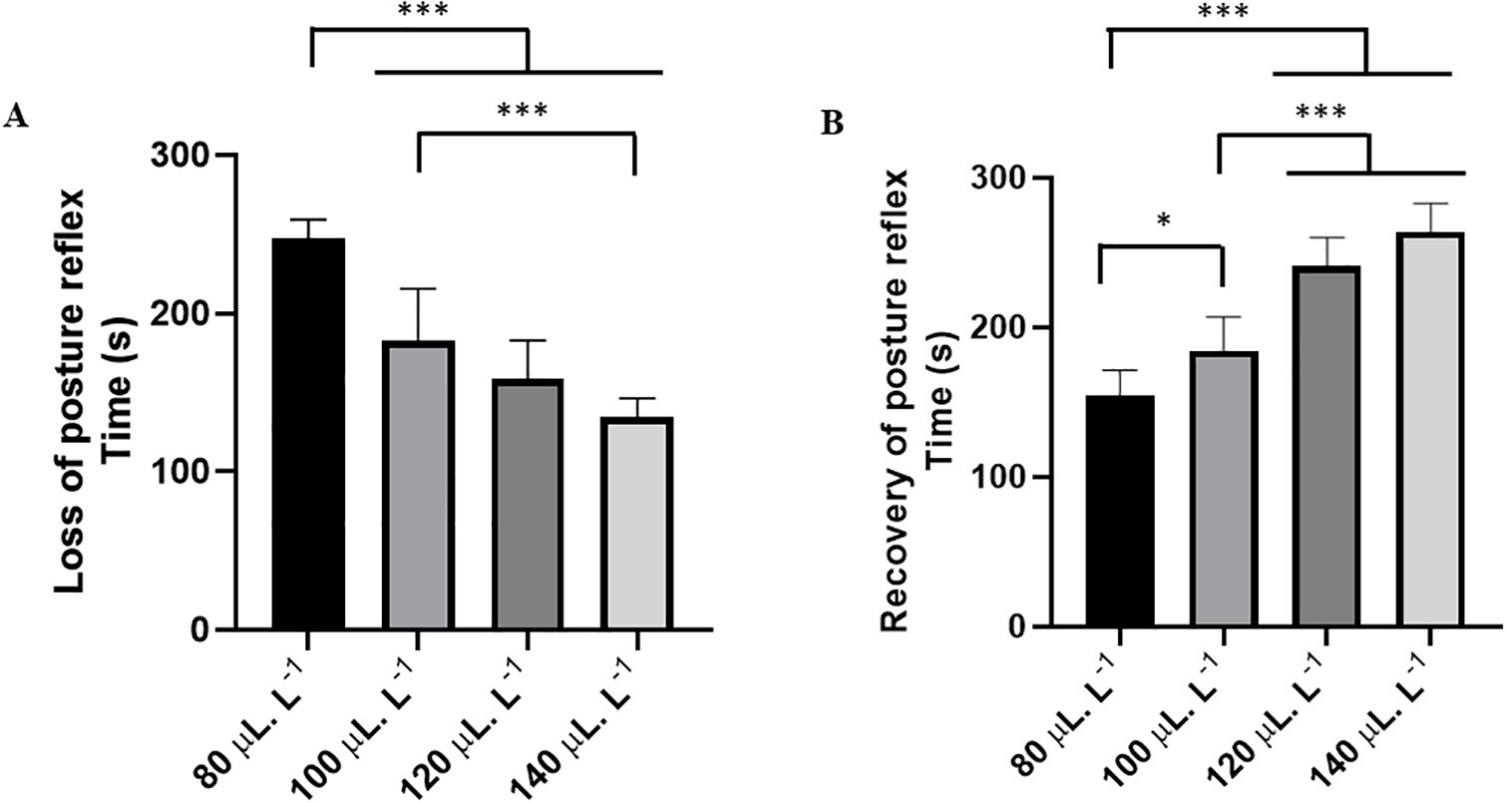
Mean latencies (s) for the loss of postural reflex in *Colossoma macropomum* during 5-minute immersion baths with different concentrations of *Lippia alba* essential oil (LAEO) (A). Recovery of postural reflex after exposure to different concentrations of LAEO (B). (ANOVA followed by Tukey’s test; *p<0.05, **p<0.01, and ***p<0.001).

The behavioral analysis showed that LAEO caused a concentration-dependent loss of postural reflex. In this sense, the higher the dose, the shorter the latency for the onset of postural reflex loss, and a small increase in concentration resulted in a faster induction. However, animals subjected to treatments of 120 and 140 μL.L^-1^ showed no difference in the time to induction (p = 0.1178). Fish exposed to 80 μL.L^-1^ had a mean time to present postural loss of 247.1 ± 12.35s, which was higher than the other groups. The group treated with 100 μL.L^-1^ (182.9 ± 32.72s) had a longer induction time than the 140 μL.L^-1^ group (134.2 ± 12.13s). Fish treated with 100 μL.L^-1^ were similar to the groups treated with 120 μL.L^-1^ (158.3 ± 24.42s) (p = 0.10820) (Fig 1A).

The recovery of the postural reflex in the group exposed to 80 μL.L^-1^ occurred in 154.1 ± 17.34 s, which was shorter than the other groups: 100 μL.L^-1^ (184.4 ± 22.19 s), 120 μL.L^-1^ (240.8 ± 19.19 s), and 140 μL.L^-1^ (263.6 ± 19.38 s). The groups treated with 120 and 140 μL.L^-1^ were similar (p = 0.0851) (Fig 1B).

**Fig 2:**
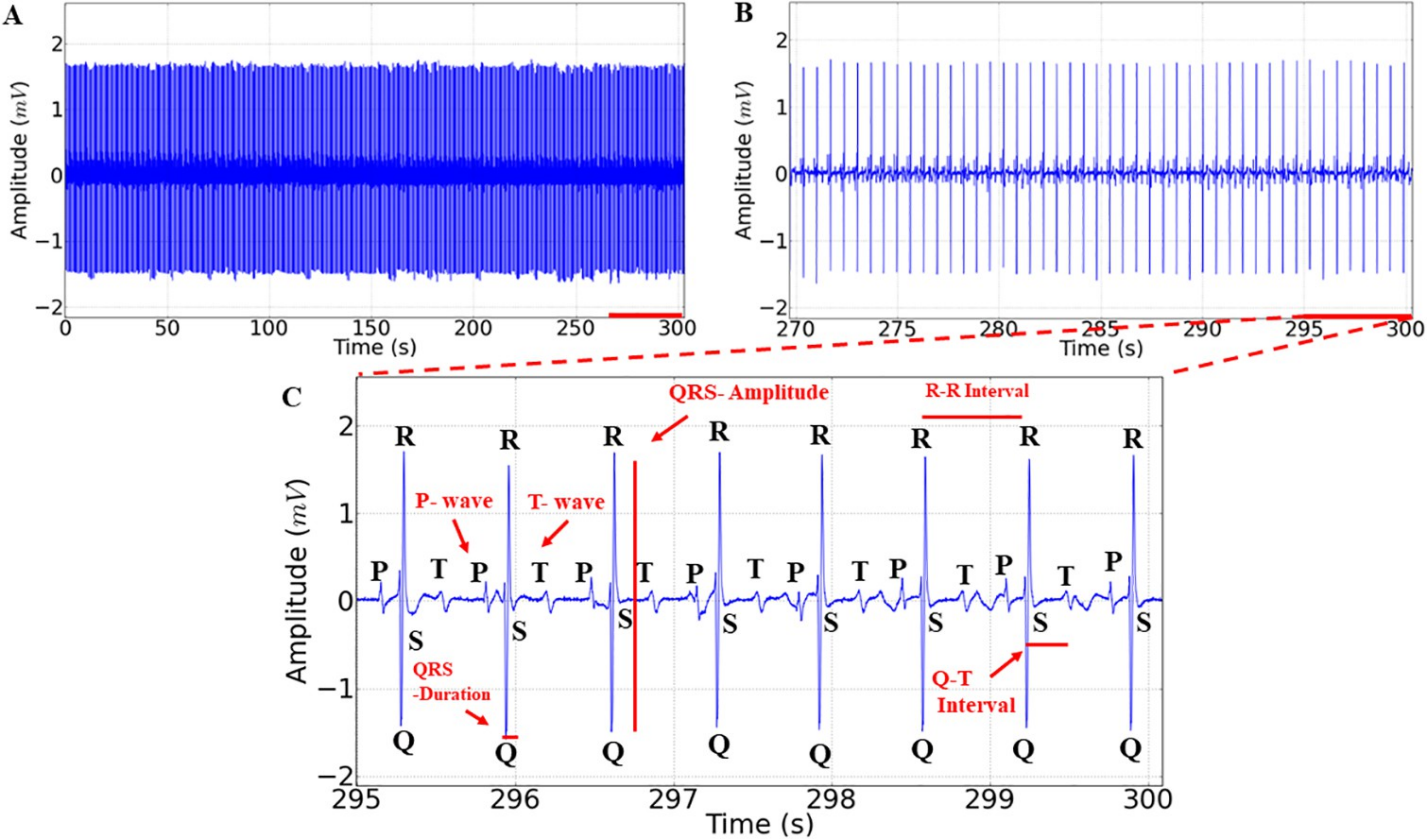
Electrocardiographic recording showing cardiac activity of the control group in juvenile *Colossoma macropomum* (A), amplification of the last 30 seconds of the 5-minute recording (270 to 300s) (B), the graph elements evaluated in the study demonstrating the P wave, QRS complex, and T wave, and variables such as heart rate (bpm), amplitude (mV), R-R, P-Q, and Q-T intervals (ms), and QRS complex duration (ms), are indicated in red (C).

Cardiac activity between the control and vehicle groups (not shown) was similar (p = 0.9998); therefore, we considered only the control group for comparisons between treatments. The control group exhibited a mean frequency of 91.78 ± 4.73 bpm, with sinus rhythm, and all cardiac elements were present in the electrocardiogram (Fig 2A). In a 30-second amplification, rhythmic cardiac activity is demonstrated, and the elements of the *Colossoma macropomum* normal activity graph can be observed, including the P wave, QRS complex, and T wave, allowing for the evaluation of intervals during immersion in LAEO and its recovery. Thus, we can identify atrial activity represented by P waves, ventricular activity by QRS complexes, and ventricular repolarization by T waves (Fig 2B and 2C). The graph elements that allowed for data evaluation are indicated in Fig 2C.

**Fig 3:**
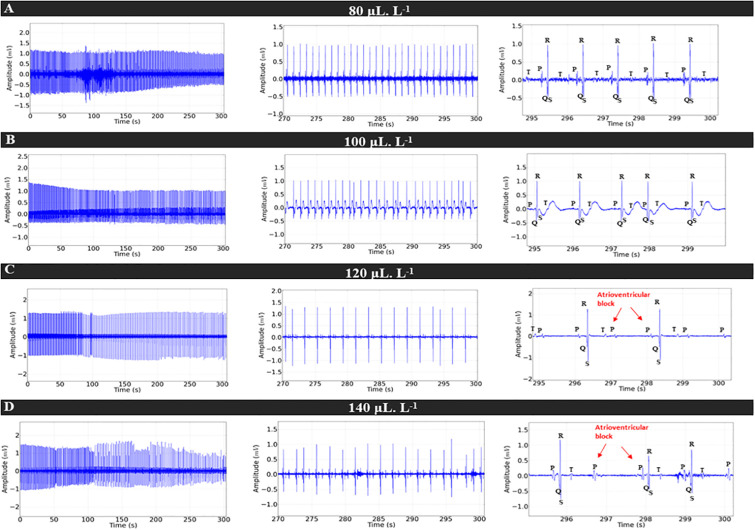
Cardiac activity in juvenile *Colossoma macropomum* during immersion bath in different concentrations of LAEO (left). Amplification of the recording in the last 30 seconds (270–300 s) (center), showing the amplification of the final 5 s of the recording (295–300 s) for identification of cardiac deflections (right), for treatment concentrations 80 μL.L^-1^ (A), 100 μL.L^-1^ (B), 120 μL.L^-1^ (C), and 140 μL.L^-1^ (D).

During treatment with LAEO at concentrations of 80 μL.L^-1^, 100 μL.L^-1^, 120 μL.L^-1^, and 140 μL.L^-1^, the ECG showed a concentration-dependent decrease in cardiac excitability (Fig 3A–3D). However, at higher treatment concentrations (120 μL.L^-1^ and 140 μL.L^-1^), a pronounced decrease in cardiac activity was observed. Moreover, there was atrioventricular blockage at these treatment concentrations, as evidenced by repeated P waves without the presence of the QRS complex, indicating non-sinus rhythm (Fig 3C and 3D). Heart rate decreased by 32.44% in the group treated with 80 μL.L^-1^ and by 33.41% in fish exposed to 100 μL.L^-1^ compared to the control. Fish receiving 120 μL.L^-1^ and 140 μL.L^-1^ exhibited more intense bradycardia compared to the control, with a respective decrease in cardiac function of 61.25% and 62.71% (Fig 3).

**Fig 4:**
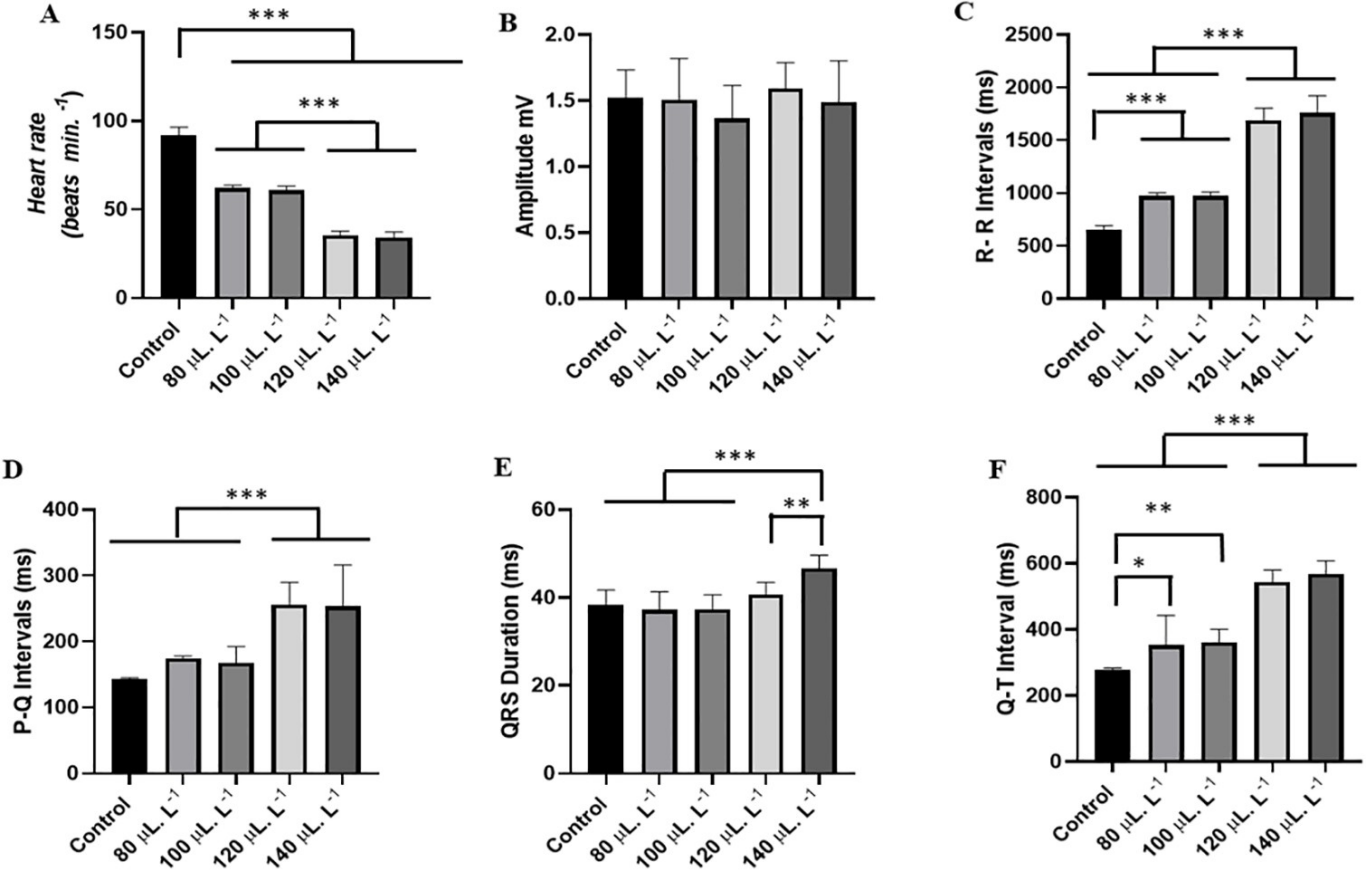
Mean values of heart rate in beats per minute (bpm) during immersion baths with LAEO treatments of 80 μL.L^-1^, 100 μL.L^-1^, 120 μL.L^-1^, and 140 μL.L^-1^ (A), mean values of QRS complex amplitude (mV) (B), mean values of R-R intervals (ms) (C), P-Q intervals (ms) (D), QRS complex duration (ms) (E), and Q-T intervals (ms) (F). (ANOVA followed by Tukey’s test; *P<0.05, **P<0.01, ***P<0.001; n = 9).

Heart rate was significantly affected by increasing concentrations of LAEO. The control group had a mean of 91.78 ± 4.73 bpm, which was higher than the other groups. The group treated with 80 μL.L^-1^ had a mean of (62.00 ± 1.73 bpm) and was similar to the group treated with 100 μL.L^-1^ (61.11 ± 2.02 bpm) (p = 0.9693), while the group treated with 120 μL.L^-1^ (35.56 ± 2.40 bpm) was similar to the group treated with 140 μL.L^-1^ (34.22 ± 3.07 bpm) (p = 0.8774) (Fig 4A).

The mean QRS complex amplitude of the control group was 1.52 ± 0.21 mV, which was similar to the other groups. The group treated with 80 μL.L^-1^ (1.50 ± 0.31 mV), the group treated with 100 μL.L^-1^ (1.36 ± 0.24 mV), the group treated with 120 μL.L^-1^ (1.59 ± 0.19 mV), and the group treated with 140 μL.L^-1^ (1.49 ± 0.31 mV) were similar (F(4,45) = 0.9945; p = 0.4204) (Fig 4B).

The mean R-R interval of the control group was 656.6 ± 34.51 ms and was lower compared to the other groups. The group treated with 80 μL.L^-1^ had a mean R-R interval of 971 ± 29.28 ms, which was similar to the group treated with 100 μL.L^-1^ (974.4 ± 34.68 ms) (p = 0.9999). The groups treated with 120 μL.L^-1^ (1689 ± 113.0 ms) were similar to the group treated with 140 μL.L^-1^ (1762 ± 160.1 ms) (p = 0.4503) (Fig 4C).

The mean P-Q interval for the control group was 143.2 ± 1.92 ms and was similar to the groups treated with 80 μL.L^-1^ (175.2 ± 1.92 ms) and 100 μL.L^-1^ (167.3 ± 25.31 ms) (p = 0.2868). The groups treated with LAEO at concentrations of 120 μL.L^-1^ (255.7 ± 34.58 ms) and 140 μL.L^-1^ (253.6 ± 62.76 ms) were similar (p = 0.9999) (Fig 4D).

The mean duration of the QRS complex for the control group during induction was 38.32 ± 3.52 ms, which was similar to the groups treated with 80 μL.L^-1^ (37.22 ± 4.17 ms), 100 μL.L^-1^ (37.33 ± 3.31 ms), and 120 μL.L^-1^ (40.67 ± 2.83 ms) (p = 0.5514). The group treated with 140 μL.L^-1^ of LAEO (46.67 ± 2.95 ms) was higher than the other groups (Fig 4E).

For the control group, the mean Q-T interval during induction was 278.7 ± 4.79 ms, which was lower than the other groups. The groups treated with 80 μL.L^-1^ (352.1 ± 90.78 ms) and 100 μL.L^-1^ (362.4 ± 38.68 ms) were similar (p = 0.9921). The 120 μL.L^-1^ group (543.3 ± 36.82 ms) and 140 μL.L^-1^ group (569.2 ± 38.43 ms) were similar (p = 0.8084) (Fig 4F).

**Fig 5:**
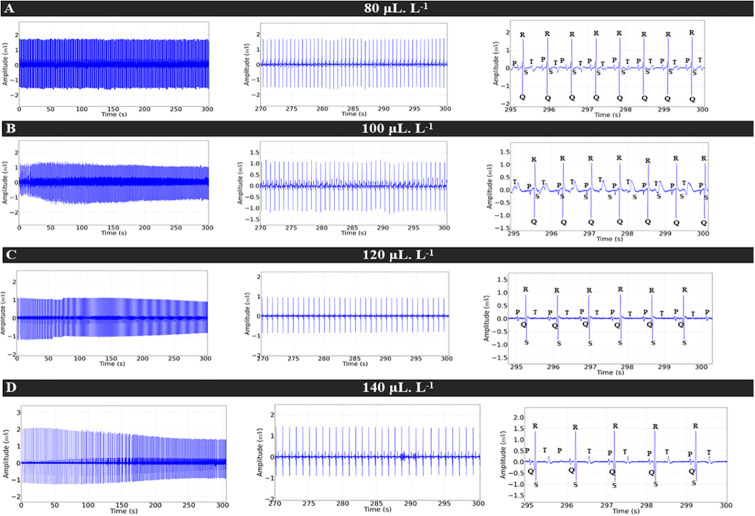
Cardiac activity in juvenile *Colossoma macropomum* during recovery after immersion baths with different concentrations of LAEO (left). Amplification of the recording in the last 30 seconds (270–300 s) (center), showing the magnification of the last 5 seconds of the recording (295–300 s) for identification of cardiac events (right), during the recovery period after immersion baths with the following concentrations of LAEO: 80 μL.L^-1^ (A), 100 μL.L^-1^ (B), 120 μL.L^-1^ (C), and 140 μL.L^-1^ (D).

During the recovery from LAEO at 80 μL.L^-1^, 100 μL.L^-1^, 120 μL.L^-1^, and 140 μL.L^-1^, a slow reversibility was observed, with sinus bradycardia being more pronounced in the group treated with the highest concentration, indicating that recovery was slow and concentration-dependent (Fig 5). The increase in heart rate gradually approached that of the control group. Accordingly, for the group treated with 80 μL.L^-1^, the heart rate value relative to the control group was 90.79% (Fig 5A). For the other groups, the values were 92.49% for 100 μL.L^-1^ (Fig 5B), 91.04% for 120 μL.L^-1^ (Fig 5C), and 70.46% for 140 μL.L^-1^ (Fig 5D). No arrhythmias were observed during the recovery period, and the reversal of LAEO effects on the heart was gradual and dependent on the concentration used (Fig 5).

**Fig 6:**
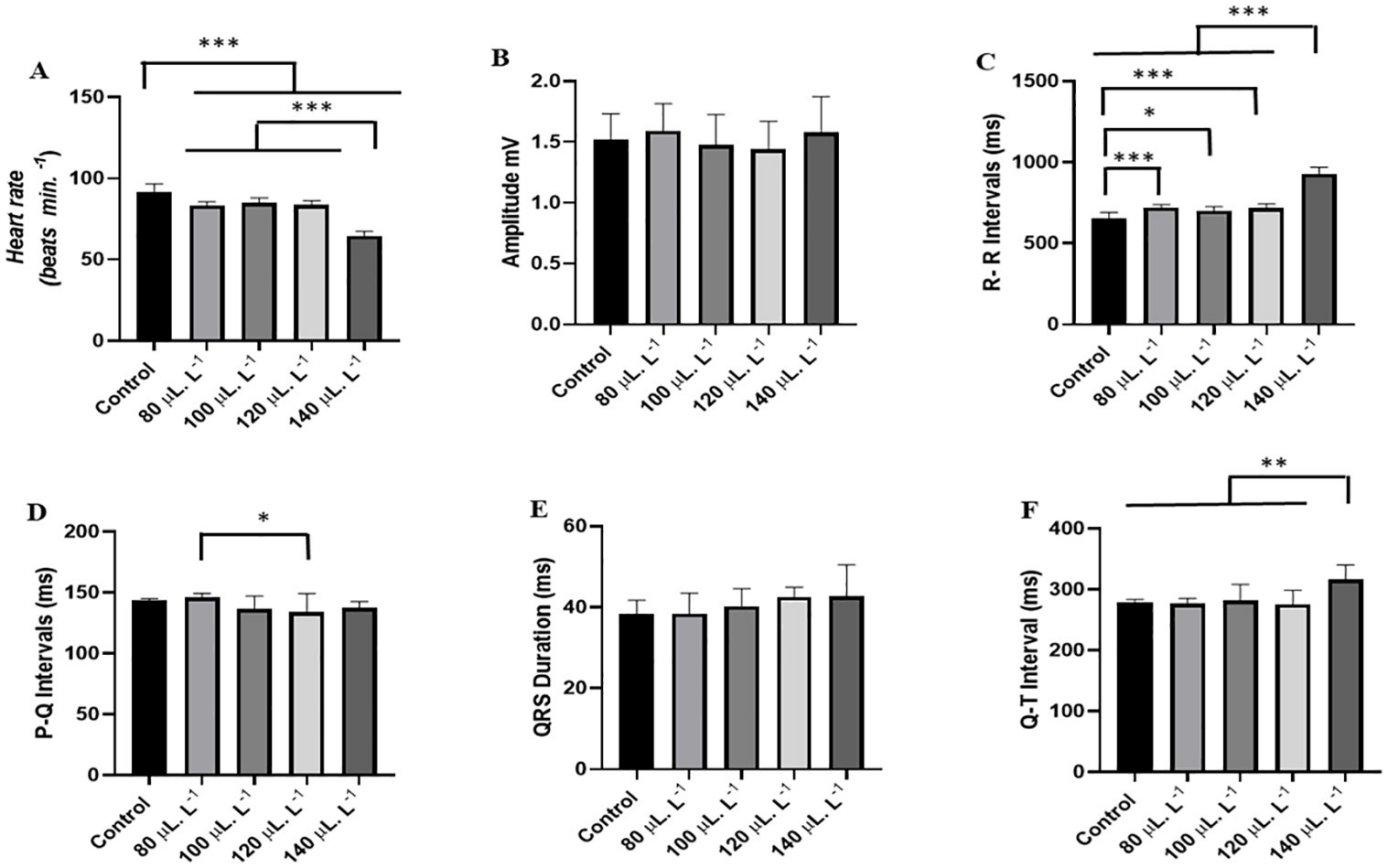
Mean values of cardiac parameters during recovery from exposure to different concentrations of LAEO: 80 μL.L^-1^, 100 μL.L^-1^, 120 μL.L^-1^, and 140 μL.L^-1^. Mean values of heart rate (bpm) (A); mean values of QRS complex amplitude (mV) (B); mean values of R-R interval (ms) (C); P-Q intervals (ms) (D); QRS complex duration (ms) (E); and Q-T interval (ms) (F). (ANOVA followed by Tukey’s test; *P<0.01, **P<0.001, ***P<0.0001; n = 9).

During the recovery period, the control group exhibited an average heart rate of 91.78 ± 4.73 bpm, which was higher than the other groups: the 80 μL.L^-1^ group with an average of 83.33 ± 2.23 bpm, the 100 μL.L^-1^ group with 84.89 ± 3.01 bpm, the 120 μL.L^-1^ group with 83.56 ± 2.78 bpm, and the 140 μL.L^-1^ group with 64.67 ± 2.82 bpm. The groups treated with 80 μL.L^-1^, 100 μL.L^-1^, and 120 μL.L^-1^ were similar (p = 0.8447) (Fig 6A).

The amplitude of the QRS complex during recovery for the control group was 1.52 ± 0.21 mV, which was similar to the other groups: 80 μL.L^-1^ (1.59 ± 0.22 mV), 100 μL.L^-1^ (1.47 ± 0.23 mV), 120 μL.L^-1^ (1.43 ± 0.23 mV), and 140 μL.L^-1^ (1.57 ± 0.29 mV) (F (4, 45) = 0.7171; p = 0.584) (Fig 6B).

The mean R-R interval during recovery for the control group was 656.6 ± 34.51 ms, which was shorter than that of the other groups. The 80 μL.L^-1^ group with 720.0 ± 19.69 ms, the 100 μL.L^-1^ group with 702.0 ± 24.22 ms, and the 120 μL.L^-1^ group with 718.4 ± 24.66 ms were similar (p = 0.7043). The group treated with 140 μL.L^-1^ had a mean R-R interval of 929.0 ± 40.88 ms, which was longer than that of the other groups (Fig 6C).

The P-Q interval during recovery for the control group was 143.2 ± 1.92 ms, which was similar to the 80 μL.L^-1^ group (146.1 ± 3.10 ms), the 100 μL.L^-1^ group (138.6 ± 15.39 ms), the 120 μL.L^-1^ group (136.6 ± 10.53 ms), and the 140 μL.L^-1^ group (137.2 ± 5.31 ms) (F(4, 40) = 3.044; p = 0.0279) (Fig 6D).

The duration of the QRS complex during recovery was similar across all groups: the control group (38.22 ± 3.52 ms), the group treated with 80 μL.L^-1^ (38.44 ± 5.05 ms), 100 μL.L^-1^ (40.22 ± 4.41 ms), 120 μL.L^-1^ (42.56 ± 2.35 ms), and 140 μL.L^-1^ (42.67 ± 7.79 ms) (F(4, 40) = 1.67; p = 0.175) (Fig 6E).

During the recovery period, the Q-T interval for the control group was 278.7 ± 4.79 ms, similar to that of fish exposed to 80 μL.L^-1^ (276.4 ± 8.77 ms), 100 μL.L^-1^ (281.9 ± 26.42 ms), and 120 μL.L^-1^ (275.0 ± 23.64 ms) (p = 0.9448). The group treated with 140 μL.L^-1^ (316.2 ± 23.96 ms) had a longer Q-T interval compared to the other groups (Fig 6F).

## Discussion

In recent years, the use of essential oils as an alternative to synthetic anesthetic agents has been gaining ground in fish farming. Anesthetic agents such as Aqui-S, which contains isoeugenol as its active ingredient, have already been approved for aquaculture application in some countries [16,17]. However, there is a noticeable search for other anesthetic agents within the scientific community. The essential oil of *L*. *alba* is recognized for its anesthetic capacity through the modulation of the GABAergic system, as previosly suggested [8]. This essential oil exhibits great variability in its constituents, which is not only related to genetic factors but also to biotic and abiotic factors, typically classified by its major compound, with the most common being citral, linalool, and carvone [18,19]. The study performed by Souza et al. (2018) [15] investigated the application of two chemotypes of *L*. *alba* essential oil (citral and linalool) as an anesthetic agent in *Rhamdia quelen*. Their results showed that the citral chemotype was stressful for this species, suggesting the use of the linalool chemotype, as it did not cause major alterations in the hypothalamus-pituitary-interrenal (HPI) axis when administered. Therefore, for the purpose of investigating safety and animal welfare, as well as possible side effects, our study was conducted with the linalool chemotype.

Our study confirmed anesthetic induction using juveniles tambaqui with *L*. *alba* within 4 minutes at the lowest concentration (80 μL.L^-1^) and under 3 minutes at higher concentrations, the recovery period remained below 5 minutes for all concentrations tested. These induction and recovery periods are recommended in the literature for short-term anesthesia, which reinforces the application of this essential [20,21]. This time interval should also be considered due to the absorption and clearance rates in fish. Over a prolonged period, the absorption rate may equal the clearance rate, resulting in a steady state that does not produce the expected anesthetic effect, which could compromise the proper induction of anesthesia [20]. Our result differed from the study conducted by Silva et al. (2019), at a concentration of 100 μL.L-1 of *L*. *alba* essential oil, the induction time reached 8 minutes, which may be explained by the difference in the weight and developmental stage of the fish. Thus, anesthetic susceptibility to a specific dosage, even among fish of the same species, depends on biological factors such as age, body weight, sexual maturity, and other physiological factors [22,23]. In agreement with this, Ribeiro et al. (2015) [24] investigated the anesthetic efficacy of eugenol in *Oreochromis niloticus* during early life stages and found that group IV (2.62 g) had longer induction times at concentrations of 50–125 mg.L⁻¹ compared to group V (11.64 g). In this context, the study by Park et al. (2018) [25] investigated the anesthetic efficacy of eugenol in juvenile and adult *Epinephelus akarra*, finding that the ratio of recovery to induction time was higher in juveniles compared to adults. Therefore, the life stage and weight of the animal undergoing anesthesia must be considered when determining the appropriate concentration of the anesthetic agent.

Furthermore, electrophysiological evaluations have been emphasized in anesthetic monitoring by several authors [26–28]. This is the first work to report the electrocardiographic evaluation of juveniles tambaqui subjected to *L*. *alba* essential oil. Our results confirm a reduction in cardiac activity of 32.44% to 62.71% during the anesthetic induction process in this species. On the other hand, a previous study that used menthol as an anesthetic agent in tambaqui recorded a significant decrease in heart rate even at lower concentrations than those tested in this work [26]. Additionally, the study carried out by Araújo et al. (2023) [29], which subjected tambaquis to anesthetic baths with geraniol and citronellol, recorded a significant reduction in QRS complex amplitude during electrocardiography, especially when using citronellol, which was not observed when using *L*. *alba* essential oil regardless of the concentration tested. This decrease in amplitude recorded in the electrocardiogram is related to the loss of cardiac muscle contractility. Thus, the loss of excitability tissue resulting from sodium channel blockade can result not only in conduction deceleration but also in excitability, reflecting the reduction in amplitude and prolongation of QRS [30]. Our work did not register this decrease in QRS complex amplitude, the animals subjected to the anesthetic presented amplitudes similar to the control.

Moreover, the present study observed the onset of atrioventricular block at higher concentrations of *L*. *alba* essential oil. This event has been reported in other studies with tambaquis using both synthetic anesthetics and plant-derived compounds. Atrioventricular block is characterized by irregular conduction of the action potential between the atrium and ventricle, resulting in dyssynchrony between these chambers [31]. A previous study demonstrated the occurrence of advanced cardiac blocks was preceded by an increase in the P-R interval and the presence of a non-conducted P wave occurring during or before the T wave of the preceding QRS complex [32]. Thus, these findings suggest that the ventricle had not yet completed repolarization when the atrium contracted again, it is proposed that the 2:1 atrioventricular block results from a prolonged ventricular refractory period that exceeds the sinus rate. In the present study, the onset of second-degree atrioventricular block was observed, along with prolongation of the P-Q, QRS, and Q-T intervals at concentrations of 120 and 140 μL.L⁻¹, leading to potential dyssynchrony in conduction between the atrium and ventricle. Additionally, Araújo et al. (2023) indicated a potential atrioventricular block in their experiment due to the prolongation of the QRS complex, RR, and QT intervals. Souza et al. (2024) [33] reported atrioventricular block in juveniles of tambaqui subjected to immersion baths with tricaine at higher concentrations (200 and 250 mg.L⁻¹). In contrast, some studies with tambaqui using lidocaine hydrochloride and menthol as anesthetic agents did not record occurrences of atrioventricular block [26,34]. Therefore, it is evident that this species is highly susceptible to cardiac alterations, and exploring various anesthetic agents and their concentrations is necessary to ensure the safety and well-being of the animal during the anesthetic process.

Despite the cardiac events during anesthetic induction, the recovery process showed reversibility of these parameters to normal levels. It was noticeable that at the highest concentration of *L*. *alba* essential oil, some parameters remained slightly below those of the control group, such as heart rate and consequently the R-R interval. Furthermore, the Q-T interval, which relates to ventricular activity, remained altered at the concentration of 140 μL.L⁻¹. However, the animals had a smooth recovery without alterations in the electrocardiogram trace. In contrast, a prior study identified P wave apiculation during anesthetic recovery using *Nepeta cataria* essential oil at a concentration of 200 μL.L^-1^, this alteration indicates atrial overload in blood pumping to the ventricle [35]. Thus, the essential oil tested in this study allows for the reversibility of cardiac parameters to normal levels during the anesthetic recovery period.

In conclusion, *Lippia alba* essential oil proved to be an efficient and reversible anesthetic during the conducted analyses. However, the evaluation of cardiac function showed that with the deepening of the anesthetic plan, there were alterations in the morphographic elements, indicating changes in ventricular function, with the presence of atrioventricular block during treatment with higher concentrations of LEOA. In this sense, *Lippia alba* essential oil proves to be safe at doses between 80 and 100 μL.L^-1^ to provide short-term anesthesia without major risks to hemodynamics in juvenile tambaquis.
